# Assessment of faecal glucocorticoid metabolite excretion in captive female fishing cats (*Prionailurus viverinus*) in Thailand

**DOI:** 10.1093/conphys/cow021

**Published:** 2016-06-02

**Authors:** Jaruwan Khonmee, Narathip Vorawattanatham, Anuchai Pinyopummin, Chatchote Thitaram, Chaleamchat Somgird, Veerasak Punyapornwithaya, Janine L. Brown

**Affiliations:** 1 Department of Veterinary Bioscience and Veterinary Public Health, Faculty of Veterinary Medicine, Chiang Mai University, Chiang Mai 50100, Thailand; 2 Veterinary, Conservation and Research Section, Animal Management Division, Chiang Mai Night Safari, Chiang Mai 50100, Thailand; 3 Department of Large Animal and Wildlife Clinical Sciences, Faculty of Veterinary Medicine, Kasetsart University, Kamphaeng Saen 10900, Thailand; 4 Department of Companion Animal and Wildlife Clinic, Faculty of Veterinary Medicine, Chiang Mai University, Chiang Mai 50100, Thailand; 5 Excellent Center for Veterinary Public Health, Faculty of Veterinary Medicine, Chiang Mai University, Chiang Mai 50100, Thailand; 6 Center for Species Survival, Smithsonian Conservation Biology Institute, Front Royal, VA 22630, USA

**Keywords:** Adrenal function, fishing cat, glucocorticoid metabolites, non-invasive hormone monitoring, seasonality

## Abstract

There is little information on the endocrinology of fishing cats (*Prionailurus viverinus*), an endangered species in Southeast Asia, especially that pertaining to adrenal function. This study characterized faecal glucocorticoid metabolites in female fishing cats housed at Chiang Mai Night Safari to investigate seasonal and age relationships in hormone patterns. Faecal samples were collected 3 days/week for 1 year from seven females ranging in age from 4.5 to 9.6 years. A corticosterone enzyme immunoassay was validated for fishing cats by showing increases (∼60%) in faecal glucocorticoid immunoactivity above pre-treatment baseline levels within 1–2 days after an adrenocorticotrophic hormone injection. Faecal glucocorticoid metabolite concentrations were not related to age (*P* > 0.05), but there was a seasonal effect, with concentrations being higher (*P* < 0.05) during the winter (1.54 ± 0.04 µg/g) and rainy season (1.43 ± 0.04 µg/g) compared with the summer (1.22 ± 0.05 µg/g). Significant relationships were found between faecal glucocorticoids and rainfall (positive) and day length (negative), but not a temperature–humidity index. This is the first study to assess adrenal steroidogenic activity in female fishing cats, and we found that glucocorticoid metabolite production was influenced by seasonal factors, but not by age. We conclude that weather patterns should be taken into consideration in future studies of glucocorticoid activity in this endangered species, especially those studies aimed at improving captive management to create self-sustaining and healthy populations.

## Abstract (precis)

Assessment of fecal glucocorticoid metabolite excretion in captive female fishing cats (Prionailurus viverinus) in Thailand

## Introduction

The fishing cat (*Prionailurus viverrinus*) is a small to medium-sized felid named for its primary prey, fish (IUCN, 2010; Santymire *et al.*, 2011). As such, they are good swimmers and typically inhabit swamps and marshy areas, oxbow lakes, reed beds along rivers and streams, tidal creeks and mangrove areas (Norwell and Jakson, 1996; Menon, 2003; Santymire *et al.*, 2011). Owing to their association with wetlands, fishing cats are widely, although discontinuously, distributed throughout Asia, including India, Bhutan, Cambodia, Thailand, China, Myanmar, Sumatra and Java (Lekagul and McNeely, 1988; Norwell and Jakson, 1996; Kanchanasakha *et al.*, 1998; Francis, 2001; IUCN, 2010; Santymire *et al.*, 2011). The species is listed as ‘endangered’ on the International Union for Conservation of Nature (IUCN) Red List (IUCN, 2010) and is included in the Wild Animal Reservation and Protection Act of Thailand Act B.E. 2535 (1992) (National Park, Wildlife and Plant Conservation, 2015). In Thailand, this species is found exclusively in protected areas and, as in other parts of Asia, is found near water, where they feed on fish, crabs, rodents, birds and hard-shelled freshwater mollusks (Lekagul and McNeely, 1988; Francis, 2001). The size of the wild fishing cat population globally, and specifically in Thailand, is unknown, but numbers are believed to be decreasing as a result of habitat loss and wetland destruction, water pollution, pesticide use and poaching (Norwell and Jakson, 1996; IUCN, 2010). As a result, this is a priority species of the Zoological Parks Organization and Chiang Mai Night Safari of Thailand for *ex situ* propagation.

Zoos are playing an increasingly important role in conservation of threatened species through captive breeding programmes (Conde *et al.*, 2011), with success being closely linked to knowledge of species biology and natural history. Such information in fishing cats is limited and includes studies of behaviour (Lekagul and McNeely, 1988; Francis, 2001), a single study of gonadal hormones in males and females (Santymire *et al.*, 2011) and a characterization of seminal traits and semen cryopreservation (Thiangtum *et al.*, 2006). Births and ovarian cycle activity have been observed throughout the year in North America (Swanson, 2007; Santymire *et al.*, 2011); however, in the coastal wetlands of northeastern India, peak mating activity occurs in January and February, with births observed primarily in March–May (Norwell and Jakson, 1996). Fishing cats reach sexual maturity at ∼15 months of age and can live up to 10 years in captivity. Gestation lasts 63–70 days, and females give birth to two or three kittens (Mellen, 1991). Global efforts to maintain healthy, self-sustaining *ex situ* populations of fishing cats have largely been hindered by problems associated with poor reproduction, behavioural incompatibility, aggression of males towards females, limited founder numbers and lack of information on basic husbandry needs (Santymire *et al.*, 2011). In Thailand, there are ∼30 fishing cats managed by the Zoological Parks Organization, and although reproduction has occurred in some facilities, overall rates are low. At the Chiang Mai Night Safari, where this study was conducted, breeding success has been inconsistent. Understanding how captive management affects species biology, particularly related to reproduction and welfare, could lead to improved husbandry protocols to manage this species better.

There are a variety of potential stressors in zoo environments, including inadequate housing, inappropriate social management and poor husbandry (Horton *et al.*, 1991; Mellen, 1991; Perkins, 1992; Carlstead and Shepherdson, 1994; Saito *et al.*, 1996; Wielebnowski *et al.*, 2002; Huber *et al.*, 2003; Shepherdson *et al.*, 2004; Morgan and Tromborg, 2007; Moreira *et al.*, 2007; Scarlata *et al.*, 2013; Khonmee *et al.*, 2014a), many of which are associated with increased glucocorticoid production (Liptrap, 1993; Möstl and Palme, 2002; Millspaugh and Washburn, 2004; Touma and Palme, 2005; Brown, 2006; Khonmee *et al.*, 2014b). One method commonly used to monitor adrenal activity as it pertains to welfare in wildlife species is the analysis of glucocorticoid metabolites excreted in urine and faeces (Palme *et al.*, 1996; Schwarzenberger *et al.*, 1996; Brown, 2006). In felids, because urine collection is difficult and glucocorticoids are primarily excreted in faeces (Brown, 2006), most non-invasive assessments of adrenal function are based on faecal analyses (Huber *et al.*, 2003; Millspaugh and Washburn, 2004; Brown, 2006). However, adrenocortical responses to various stressors may be confounded by daily and seasonal variations in basal hormone production (Millspaugh and Washburn, 2004; Allwin *et al.*, 2014; Khonmee *et al.*, 2014a, b; Parnell *et al.*, 2014). In addition, age can influence glucocorticoid secretion (Vallée *et al.*, 1999; Downs *et al.*, 2008).

The aim of this study was to investigate the influence of season and age on glucocorticoid excretion in fishing cats and determine whether these factors could potentially confound assessments of adrenal function. This information will be key to interpreting glucocorticoid data in association with studies designed to improve management of an endangered species of national importance to Thailand.

## Materials and methods

### Ethics statement

This study was approved by the Faculty of Veterinary Medicine Chiang Mai University Animal Care and Use Committee (FVM-ACUC; permit number R11/2558). Permission to conduct the study from Chiang Mai Night Safari was obtained from the staff veterinarian and mammal curator, who were also collaborators on the project.

### Environmental data

There are three major seasons in Thailand: summer (16 February–15 May), rainy (16 May–15 October) and winter (16 October–15 February). Information on average temperature (in degrees Celsius), amount of rainfall (in millimetres per day), humidity (expressed as a percentage) and day length (in hours) in each month during the study period was obtained from The Northern Meteorological Center, Meteorological Department, Ministry of Information and Communication Technology, Chiang Mai, Thailand (Thai Meteorological Department, 2015). A thermal–humidity index (THI) was calculated based on air temperature and relative humidity using the following formula (Herbut and Angrecka, 2012): THI = (1.8 × *T*_db_ + 32) − (0.55 − 0.0055 × RH) × (1.8 × *T*_db_ − 26), where *T*_db_ is the dry bulb temperature (in degrees Celsius) and RH the relative humidity (expressed as a percentage).

### Animals and sample collection

Over the past 15 years, 30 fishing cats have been produced at the Chiang Mai Night Safari, with more than half born in July, August and September (*n* = 18). Seven of the captive-born females (7.0 ± 0.9 years of age; range, 4.5–9.6 years) were used in this study and were housed individually in off-exhibit enclosures, 2 m × 3 m × 2.5 m (*n* = 5) or 2 m × 1.5 m × 2.5 m (*n* = 2) in size. Males were kept adjacent to females in the same complex, separated by wire mesh; thus, all cats had visual, olfactory and auditory contact with conspecifics. All females were nulliparous, and no breeding introductions were conducted during the study period. The enclosures contained a concrete floor, at least one nest box and multiple branches for climbing. Animals were exposed to natural light and fed a diet of pork meat and chicken bone daily, with unlimited access to fresh water. All animals were given annual physical examinations by the staff veterinarian and were dewormed every 6 months. Females were in good health during the study period. Old faeces were removed every evening, and fresh faeces (∼30 g) were collected 3 days/week between 08.30 and 09.30 h for 1 year. Samples were stored at −20°C until processing.

### Faecal extraction

All chemicals were obtained from the Sigma Chemical Company (St Louis, MO, USA) unless otherwise stated. Wet faecal samples were dried using a conventional oven at 60°C for 5 days and stored at −20°C until extraction. Frozen dried faecal samples were thawed at room temperature, mixed well and pulverized, and 0.2 (±0.02) g of faecal powder was boiled in 5 ml of 90% ethanol:distilled water for 20 min. After centrifugation (3500***g***, 20 min), the supernatant was recovered and the faecal pellet resuspended in 5 ml of 90% ethanol:distilled water, vortexed for 1 min and recentrifuged (3500***g***, 15 min). Both supernatants were combined, dried under air and redissolved in 1 ml methanol. The extracts were stored at −20°C until further analysis. The efficiency of extraction of steroid from faeces was 91.8% based on the recovery of corticosterone standard added to dried faecal samples before extraction.

### Enzyme immunoassay

A double-antibody enzyme immunoassay (EIA) was used that relies on a polyclonal rabbit anti-corticosterone antibody (CJM006) that has been validated for quantifying glucocorticoid metabolites in faecal extracts from a wide range of avian and mammalian species, including several felids (Watson *et al*., 2013). Antiserum was produced against corticosterone-3-carboxymethyl oxime-bovine serum albumin (Steraloids, Wilton, NH, USA; Q1559-000; Vaitukaitis *et al*., 1971) and had the following tested cross-reactivities: corticosterone, 100%; desoxycorticosterone, 14.25%; progesterone, 2.65%; tetrahydrocorticosterone, 0.90%; testosterone, 0.64%; cortisol, 0.23%; prednisolone, 0.07%; 11-desoxycortisol, 0.03%; prednisone, <0.01%; cortisone, <0.01%; and estradiol, <0.01% (Watson *et al.*, 2013). Horseradish peroxidase (Sigma-Aldrich, St Louis, MO, USA; catalogue no. P8375) was coupled to corticosterone-CMO (Sigma-Aldrich) using the mixed anhydride method (Munro and Stabenfeldt, 1984). Secondary antibody-coated plates were prepared by diluting anti-rabbit IgG (10 µg/ml; catalogue no. A009; Arbor Assays, Ann Arbor, MI, USA) in coating buffer (catalogue no. X108, 20X; Arbor Assays) and adding 150 µl to each well of a 96-well microtitre plate (catalogue no. 07-200-39; Fisher Scientific, Pittsburgh, PA, USA) followed by incubation at room temperature (RT) for 15–24 h. The contents of the wells were emptied, the plates were blotted dry, and blocking solution (catalogue no. X109, 10X; Arbor Assays) was added to each well (250 µl) and incubated for 15–24 h at RT. Following incubation, the contents of the wells were emptied, and the plates were then blotted and dried at RT in a Dry Keeper (Sanplatecorp., Osaka, Japan) with loose desiccant in the bottom. After drying (humidity, <20%), plates were heat sealed in a foil bag with a 1 g desiccant packet and stored at 4°C until use.

Antibody-coated plates were brought to RT, and EIA buffer (0.1 M NaPO_4_, 0.149 M NaCl and 0.1% bovine serum albumin, pH 7.0) was added to the non-specific binding (75 µl; NSB) and maximal binding (50 µl) wells. Corticosterone standards (50 µl, range 3.9–1000 pg/well; C2505; Sigma-Aldrich, Poole, UK) and samples (50 µl, 1:200 dilution), diluted in EIA buffer, were combined with corticosterone–horseradish peroxidase (25 µl; 1:300 000 dilution) followed by addition of 25 µl of primary antibody (25 µl; 1:100 000 dilution), except for NSB wells, and incubated at RT for 1 h. Plates were washed four times with wash buffer before addition of 100 µl of TMB substrate solution (Ward Medic, Bangkok, Thailand). After incubation for 45–60 min at RT without shaking, the absorbance was measured at 620 nM (TECAN Sunrise™ microplate reader, Salzburg, Austria) until the optical density approached 0.9, at which point stop solution was added (50 µl) to each well. The absorbance was measured at 405 nM (TECAN Sunrise™ microplate reader).

The corticosterone EIA was validated for fishing cat faeces by demonstrating parallelism between serial dilutions of pooled extracts and the corticosterone standard curve (Pearson’s correlation coefficient, *r* = 0.99). Addition of unlabelled corticosterone standard to pooled faecal extracts before extraction resulted in a significant (*P* < 0.05) recovery of mass (*y* = 0.91*x* − 19.64, *R*^2^ = 0.97). Assay sensitivity was 0.078 ng/ml at 90% binding. Inter-assay coefficients of variation were 13.5% based on binding of high (30%) and low (70%) control samples. All samples were re-analysed if the duplicate coefficient of variation was >10%; thus, intra-assay coefficients of variation were <10%. Data are expressed as micrograms per gram of dry faeces.

### Adrenocorticotrophic hormone challenge

The EIA was biologically validated by means of an adrenocorticotrophic hormone (ACTH) challenge in two females. Cats were restrained in a net, followed immediately by intramuscular injection of a slow-release ACTH gel (15 IU/kg, 40 IU/ml; Wedgewood Pharmacy, Sewell, NJ, USA) in July (rainy season). Faeces were collected from each cat during the daily husbandry routine. Both females defecated about once per day, but occasionally no faeces were found. Five faecal samples were collected during the week before (including day 0) and five in the week after the ACTH injection for both cats (*n* = 10 samples each). Faecal material was stored frozen (−20°C) in plastic bags until processing and analysis.

### Statistical analyses

For the ACTH challenge, to establish pre-treatment baseline, values above the mean + 1.5SD (including day 0) were removed and recalculated iteratively until none exceeded that. Adrenocortical responses to ACTH were expressed as a percentage of the pre-treatment baseline, with the baseline being equivalent to 100%. Increases post-ACTH were significant if they exceeded the baseline mean + 1.5SD (Young *et al*., 2004).

Descriptive data of glucocorticoid metabolite concentrations for each female were averaged by month, season and overall and were presented as the mean ± SEM. Individual data were analysed using a generalized least-squares (GLS) method with R version 3.2.2 (R Development Core Team, 2015), and *nlme* package 3.1-122 (Pinheiro *et al.*, 2015). We constructed the model using only THI, rainfall and day length, and excluded temperature and humidity because of potential collinearity. Season parameters and age were defined as fixed effects. Individual fishing cat was defined as a random effect. For GLS modelling, the Akaike information criterion was determined from models with different covariance structures, including compound symmetry, autoregressive process of order 1 (ar1) and general correlation matrix with no structure. The Akaike information criterion from ar1 was the lowest value, indicating the best-fitted model. Tukey’s test was used for multiple comparisons when mean differences were significant. Residuals from the fitted model were tested for normality and homogeneity of variance assumption by plotting standardized residuals vs. quantiles of standard normal (QQ normality graph) and plotting standardized residuals vs. fitted values, respectively. The plot indicated no violation for both assumptions, which revealed that the transformation of glucocorticoid metabolite data was not necessary.

Associations between glucocorticoid metabolite concentrations and weather data were determined by GLS. The independent factors were THI, rainfall and day length. Temperature and humidity were not included in the model because they co-varied with THI. The comparison of means for environmental data (temperature, rainfall, day length, humidity and THI) by season was determined by GLS followed by Tukey’s tests. The significance level (α) was set at 0.05 for all statistical analysis.

## Results

**Figure 1:**
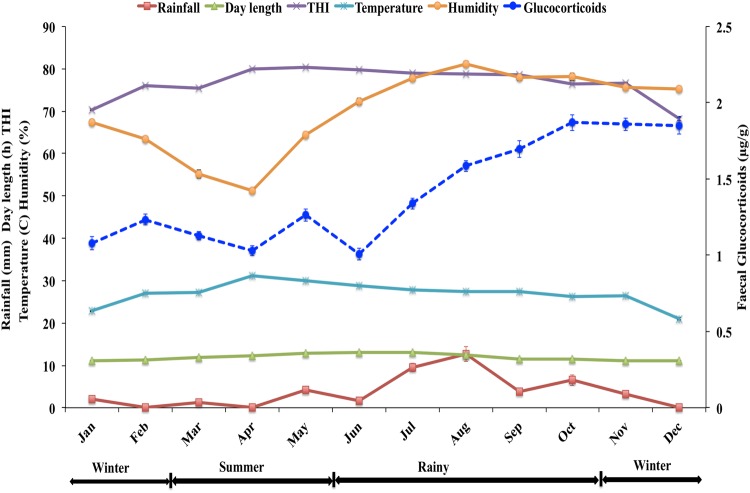
Overall mean (±SEM) monthly faecal glucocorticoid concentrations in seven female fishing cats housed at the Chiang Mai Night Safari, and weather parameters, including rainfall (in millimetres), day length (in hours) and the thermal–humidity index (THI) obtained from The Northern Meteorological Center, Meteorological Department, Chiang Mai, Thailand during the study period.

Seasonal differences in weather factors at Night Safari are shown in Table 1 and Fig. 1. As expected, average daily temperature was highest during the summer; day length, rainfall and humidity were greatest in the rainy season; and humidity was lowest in the summer (*P* < 0.05). As with rainfall and humidity, the THI was highest in the rainy season but then followed temperature, being lowest in the winter (*P* < 0.05).

**Table 1:** Mean ± SEM temperature, rainfall, day length, humidity and the thermal–humidity index across seasons at the Chiang Mai Night Safari during the study period

Season	Temperature (°C)	Rainfall (mm)	Day length (h)	Humidity (%)	THI
Summer	29.12 ± 0.13^a^	1.12 ± 0.24^b^	12.08 ± 0.03^b^	56.04 ± 0.53^c^	77.95 ± 0.16^b^
Rainy	28.01 ± 0.07^b^	6.65 ± 0.52^a^	12.48 ± 0.03^a^	75.86 ± 0.37^a^	79.08 ± 0.07^a^
Winter	24.11 ± 0.15^c^	2.33 ± 0.37^b^	11.15 ± 0.01^c^	72.94 ± 0.40^b^	72.81 ± 0.23^c^

Data were obtained from The Northern Meteorological Center, Meteorological Department, Chiang Mai, Thailand. Abbreviation: THI, thermal–humidity index. ^a^,^b^,^c^Values differ among seasons; different letters indicate differences (*P* < 0.05).

**Figure 2:**
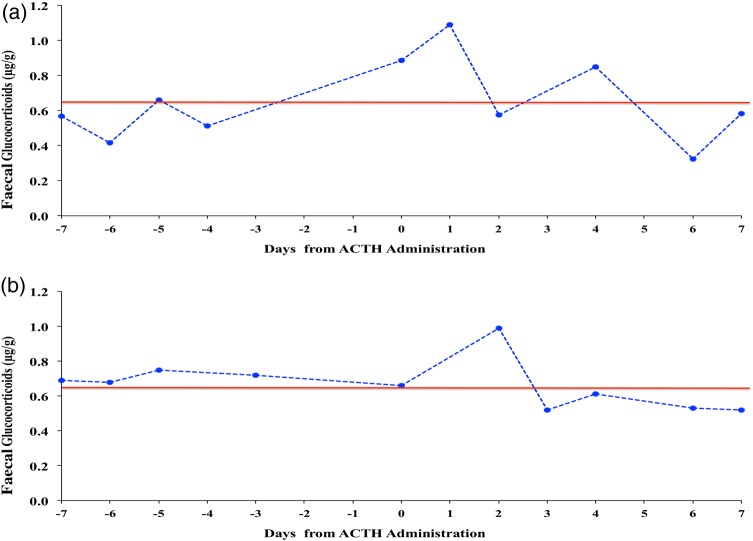
Glucocorticoid metabolites in faeces of two females, F2 (**a**) and F4 (**b**), before and after administration of exogenous adrenocorticotrophic hormone (day 0). The red line represents the baseline concentration in each female.

Faecal glucocorticoid metabolite concentrations increased within 1–2 days following ACTH administration. Pre-ACTH treatment baseline concentrations were 0.63 ± 0.06 and 0.63 ± 0.03 µg/g, and they increased to peaks of 1.09 and 0.99 µg/g after ACTH for the two treated cats, respectively (Fig. 2).

Average faecal glucocortcoid concentrations varied across the seasons, being highest in the winter and rainy season and lowest in the summer (Table 2). However, there was no effect of age on faecal glucocorticoid concentrations (*P* > 0.05).

**Table 2:** Mean ± SEM faecal glucocorticoid metabolite concentrations in seven female fishing cats across seasons at the Chiang Mai Night Safari, Chiang Mai, Thailand

Season	Glucocorticoid metabolites (µg/g)
Summer	1.15 ± 0.02^c^
Rainy season	1.42 ± 0.02^b^
Winter	1.61 ± 0.03^a^

^a^,^b^,^c^Values differ among seasons; different letters indicate differences between summer and rainy season (*P* = 0.016) and winter (*P* < 0.001), as well as between rainy season and winter (*P* = 0.044). Numerator Degree of Freedom = 2, Denominator Degree of Freedom = 1056, *F* = 11.43 (*P* < 0.001).

**Figure 3:**
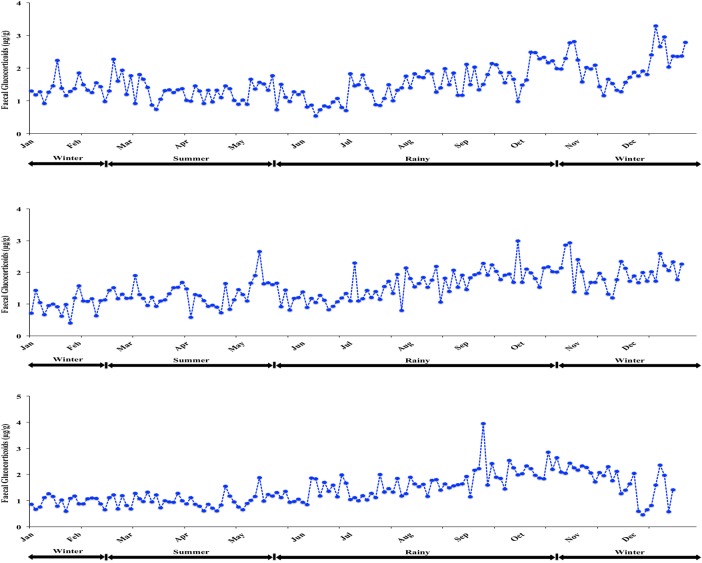
Representative longitudinal profiles of faecal glucocortcoid metabolite concentrations in inidvidual female fishing cats housed at the Chiang Mai Night Safari over a 1 year period.

Overall monthly faecal glucocorticoid concentrations for all cats combined are shown in Fig. 1. In general, glucocorticoid concentrations were low during much of the year, i.e. late winter, summer and early rainy seasons, before reaching a peak in October. This same pattern was apparent in individual females despite considerable variability from day to day, as illustrated in Fig. 3. The correlation between faecal glucocorticoid metabolite concentrations and day length was negative; it was positive with rainfall, and not significant with THI (Table 3).

**Table 3:** Generalized least-squares analysis to determine the effect of weather parameters on faecal glucocorticoid concentrations in seven female fishing cats housed at the Chiang Mai Night Safari

Effect	Estimate	SE	*t*	*P*-value
Intercept	3.756	0.470	7.999	0.001
Rainfall	0.005	0.001	3.683	0.001
Day length	−0.220	0.041	−5.336	0.001
THI	0.004	0.006	0.633	0.527

A value of *P* < 0.05 indicates statistical significance. Abbreviation: THI, thermal–humidity index.

## Discussion

This is the first study to characterize glucocorticoid metabolite excretion in female fishing cats, and confirmed an influence of seasonality, but not age, on adrenal activity. The corticosterone EIA was validated for this species by demonstrating parallelism between dilutions of faecal extracts and the standard curve, and significant recovery of exogenous corticosterone added to samples before extraction. We also saw elevations in glucocorticoid concentrations above baseline within 1–2 days after ACTH injection, a common validation used in other carnivores, including the cheetah (Terio *et al.*, 1999), clouded leopard (Wielebnowski *et al.*, 2002), domestic cat (Graham and Brown, 1996), African wild dog (Monfort *et al.*, 1998), spotted hyena (Goymann *et al.*, 1999), black-footed ferret (Young *et al.*, 2001) and carnivores (Young *et al.*, 2004). However, the ACTH response in our study was less than expected, which may have been attributable to the use of a generic product instead of ACTHAR gel. We have noted attenuated ACTH responses to this product over the past several years in other species (e.g. cheetah, maned wolf and jaguar; J. L. Brown, A. Crosier and N. Songsasen, unpublished data). Others warn against using generic ACTH products because they are not subject to regulation or quality control and are known to provide variable results (Peterson, 2011). That said, the antibody we used has been validated for assessing faecal glucocorticoid activity in >35 species, including several felids (Watson *et al*., 2013).

Adrenocorticoid responses play an important role in the seasonal and daily regulation of physiological states in many species (Smith *et al.*, 2012; Wingfield, 2012; Allwin *et al.*, 2014). In fishing cats, the seasonal effect on glucocorticoid excretion was significant, with overall concentrations being higher during the rainy season and early winter compared with the late winter and summer. Seasonal patterns in cortisol production have been observed in a number of species, tied to a variety of physiological and environmental factors. Higher glucocorticoids may be needed during more power-consuming periods of the life cycle (reproduction, migration and temperature maintenance) and to maintain catabolic function during the winter as an adaptation to colder weather (Romero *et al.*, 2000; Huber *et al.*, 2003). Such adrenal responses are often species specific, however, including whether they are adaptive or maladaptive. Heightened adrenal activity can suppress reproduction and correlate negatively with reproductive hormone production [baboon (*Papio anubis*), Sapolsky, 1985; rat, Orr and Mann, 1992; guinea pig, Fenske, 1997; mice, Dong *et al.*, 2004], whereas co-production of reproductive hormones and glucocorticoids may be associated with normal breeding seasons. For example, faecal glucocorticoid levels were closely matched to the mating period in free-ranging male muriqui monkeys (*Brachyteles arachnoides*), which corresponded to the dry season in Brazil (Strier *et al.*, 1999). Red deer (*Cervus elaphus*) showed a marked increase in faecal glucocorticoid metabolites in December and January, which followed the breeding season in September–November (Huber *et al.*, 2003). Deer mice (*Peromyscus maniculatus*) and red-backed voles (*Clethrionomys gapperi*) exhibited increased faecal glucocorticoid concentrations in late August–late September and in mid- to late September, respectively, again following the late summer, early autumn breeding seasons (Harper and Austad, 2001).

The seasonal elevation in glucocorticoid excretion in fishing cats preceded by several months, rather than followed or coincided with, the purported winter breeding season in January and February (Norwell and Jakson, 1996), including in Thailand. Food and water resources were consistent throughout the year in our study, eliminating that as a regulatory factor, but it might have been influenced by subtle changes in photoperiod, because glucocorticoid concentrations were negatively associated with day length. Increasing glucocorticoid concentrations during the late autumn/winter months, purportedly influenced by photoperiod, have been shown in other species. For example, in white-footed mice (*Peromyscus leucopus*), glucocorticoids increased during short days, suggesting photoperiod-evoked modification of the hypothalamic–pituitary–adrenal axis that might be adaptive for winter survival (Pyter *et al.*, 2007). In golden hamsters (*Mesocricetus auratus*), exposure to short days altered basal adrenal glucocorticoid secretion and corticosteroid receptors, both of which play a central role in the regulation of circadian and circannual rhythms of the hypothalamic–pituitary–adrenal axis (Ronchi *et al.*, 1998).

There also was a positive relationship with rainfall, although it is not clear whether the rainfall and day length effects were co-dependent. Faecal glucocorticoids were not significantly correlated with THI, which takes into consideration both temperature and humidity and is a measure often used to assess consequences of heat stress (Du Preez, 2000; Bouraoui *et al.*, 2002; Hansen, 2009). In felids, evaporative heat loss is largely and most efficiently facilitated by panting, although the process of sweating can by mimicked by wetting the pelage and skin in lower humidity (Young *et al.*, 2012). Our fishing cats were housed in a shaded environment and were rarely observed panting. That, and the glucocorticoid results, suggests that thermal stress was not a problem in our population.

### Conclusion

This study provides further support for non-invasive faecal glucocorticoid metabolite monitoring as a valuable tool for evaluating adrenal function in felid species (Wielebnowski *et al.*, 2002; Young *et al.*, 2004; Rozhnov *et al.*, 2010; Naidenko *et al.*, 2011; Conforti *et al.*, 2012), including the fishing cat. Further studies could include additional validations using radiolabel infusion to determine excretory routes and specific metabolism of glucocorticoid hormones (Brown *et al.*, 1994; Palme *et al.*, 2005), in addition to testing the effects of sample age, storage and extraction methods on hormone quantification (Millspaugh and Washburn, 2004). Practical application of this technique to improve fishing cat management should involve evaluations of relationships between adrenal activity and daily rhythms, body condition, reproductive status, husbandry practices and social settings. The finding of a significant seasonal effect highlights the importance of taking this into consideration when interpreting glucocorticoid data and using results to make management decisions. There is enormous potential for using this technique to improve captive management and create healthy populations of this species, something that is needed given the lack of sustainability of current captive collections.

## Funding

This work was supported by Chiang Mai University Junior Research Fellowship Program (grant number R000012700).
